# Identifying the potential transcriptional regulatory network in Hirschsprung disease by integrated analysis of microarray datasets

**DOI:** 10.1136/wjps-2022-000547

**Published:** 2023-04-17

**Authors:** Wenyao Xu, Hui Yu, Dian Chen, Weikang Pan, Weili Yang, Jing Miao, Wanying Jia, Baijun Zheng, Yong Liu, Xinlin Chen, Ya Gao, Donghao Tian

**Affiliations:** 1 Department of Pediatric Surgery, the Second Affiliated Hospital, Xi'an Jiaotong University, Xi'an, China; 2 Institute of Neurobiology, Environment and Genes Related to Diseases Key Laboratory of Chinese Ministry of Education, Xi'an Jiaotong University, Xi'an, China; 3 Department of Pulmonary and Critical Care Medicine, Peking University Third Hospital, Peking University, Beijing, China

**Keywords:** congenital abnormalities, pediatrics, neonatal screening

## Abstract

**Objective:**

Hirschsprung disease (HSCR) is one of the common neurocristopathies in children, which is associated with at least 20 genes and involves a complex regulatory mechanism. Transcriptional regulatory network (TRN) has been commonly reported in regulating gene expression and enteric nervous system development but remains to be investigated in HSCR. This study aimed to identify the potential TRN implicated in the pathogenesis and diagnosis of HSCR.

**Methods:**

Based on three microarray datasets from the Gene Expression Omnibus database, the multiMiR package was used to investigate the microRNA (miRNA)–target interactions, followed by Gene Ontology (GO) and Kyoto Encyclopedia of Genes and Genomes (KEGG) enrichment analyses. Then, we collected transcription factors (TFs) from the TransmiR database to construct the TF–miRNA–mRNA regulatory network and used cytoHubba to identify the key modules. Finally, the receiver operating characteristic (ROC) curve was determined and the integrated diagnostic models were established based on machine learning by the support vector machine method.

**Results:**

We identified 58 hub differentially expressed microRNAs (DEMis) and 16 differentially expressed mRNAs (DEMs). The robust target genes of DEMis and DEMs mainly enriched in several GO/KEGG terms, including neurogenesis, cell–substrate adhesion, PI3K–Akt, Ras/mitogen-activated protein kinase and Rho/ROCK signaling. Moreover, 2 TFs (*TP53* and *TWIST1*), 4 miRNAs (*has-miR-107*, *has-miR-10b-5p*, *has-miR-659-3p*, and *has-miR-371a-5p*), and 4 mRNAs (*PIM3*, *CHUK*, *F2RL1*, and *CA1*) were identified to construct the TF–miRNA–mRNA regulatory network. ROC analysis revealed a strong diagnostic value of the key TRN regulons (all area under the curve values were more than 0.8).

**Conclusion:**

This study suggests a potential role of the TF–miRNA–mRNA network that can help enrich the connotation of HSCR pathogenesis and diagnosis and provide new horizons for treatment.

**WHAT IS ALREADY KNOWN ON THIS TOPIC**

- Hirschsprung disease (HSCR) is one of the common neurocristopathies in children that involves a complex pathogenesis. It is difficult to develop early diagnosis of HSCR, and surgery commonly gives rise to medical complications, especially fatal enterocolitis (about 35% after surgery).

**WHAT THIS STUDY ADDS**

- A potential transcription factor–microRNA–mRNA regulatory network was identified as for the key regulons of which the receiver operating characteristic analysis revealed a strong diagnostic value in HSCR.

**HOW THIS STUDY MIGHT AFFECT RESEARCH, PRACTICE OR POLICY**

- This study suggests a transcriptional regulatory network implicated in the pathogenesis and diagnosis of HSCR, which also provides new horizons and targets for treatment.

## Introduction

Hirschsprung disease (HSCR) is one of the common neurocristopathies in children, which is characterized by aganglionosis.^1 2^ HSCR is primarily treated by surgery to eliminate the aganglionic bowel while commonly giving rise to medical complications, especially fatal enterocolitis (about 35% after surgery),^2–4^ stool leakage, anastomotic stricture, anastomotic leak with abscess, and chronic constipation. Therefore, detailed pathogenesis and effective alternatives should be developed.

At present, it is well known that the pathogenesis of HSCR is the dysfunction of enteric neural crest-derived precursors migrating through the bowel in a rostral-to-caudal direction from week 3 to week 8 of human gestation.^2^ Emerging studies have reported the effects of enteric neural crest-derived cell (ENCC) transplantation for treating the HSCR model.^5–7^ However, because of the limited proliferation, migration and large-scale apoptosis during transplantation, ENCC transplantation often tends to be an insufficient cure for HSCR.^1^ Although researchers have tried the ENCCs treated with cytokines, drugs, and signaling pathway regulators to optimize cell transplantation, it failed to completely repair the enteric nervous system (ENS).^8 9^ As supposed, HSCR is associated with at least 20 genes of more than seven chromosomal loci, involving a complex regulatory to ENCCs, but not single genetic factors.^2 10 11^ Therefore, it is necessary to explore more details of the gene expression regulatory in HSCR.

Previous studies have shown that microRNAs (miRNAs) bind on the 5′ untranslated regions of mRNAs through partial complementarity and reduce gene expression by restraining mRNA translation and/or facilitating mRNA degradation.^12^ Many miRNAs have been reported to be related to HSCR,^13–15^ such as *miRNA-206*,^16^ *miR-146b-5p*,^17^ and *miR-181a*.^18^ Like the functional genes, miRNA expression is regulated by transcription factors (TFs). Transcriptional regulatory network (TRN), demonstrating the relationship of TF–miRNA–mRNA, commonly plays roles in the regulation of gene expression and cell biological function,^19–21^ and has been reported in ENS development,^22^ neural stem cell phenotype,^20^ and cancer pathogenesis.^23^ However, the role of TRN in HSCR remains to be investigated.

In this study, we performed integrated analysis of three microarray datasets from the Gene Expression Omnibus (GEO) database, based on which a potential TF–miRNA–mRNA network was constructed. Receiver operating characteristic (ROC) analysis based on the support vector machine (SVM) method revealed a strong diagnostic value of the key TRN regulons, which can help enrich the connotation of HSCR pathogenesis and diagnosis and provide new horizons for treatment.

## Materials and methods

### Microarray datasets and processing

The mRNA and miRNA expression profiles of patients with HSCR were obtained from the GEO database (https://www.ncbi.nlm.nih.gov/geo/), which was searched using the following terms: “Hirschsprung disease” AND “microarray” AND “Homo sapiens”. The following eligibility criteria were used to include or exclude datasets and samples: (1) the dataset contained at least three patients with HSCR and three controls; (2) the colons from HSCR and normal subjects were used for microarray analysis; and (3) raw data were available in the GEO database. Detailed information of the microarray datasets is listed in table 1.

**Table 1:** Characteristics of three microarray datasets included in the study

GSE accession	Participants	Data type	Samples	Platform	Year
GSE77296	6 patients with HSCR and 3 healthy controls	miRNA microarray	Colon tissue	GPL18058	2016
GSE96854	3 patients with HSCR and 3 healthy controls	mRNA microarray	Colon tissue	GPL18943	2017
GSE98502	8 patients with HSCR and 8 healthy controls	mRNA microarray	Colon tissue	GPL22361	2018

GSE, Series in Gene Expression Omnibus database; HSCR, Hirschsprung disease; miRNA, microRNA.

The probe sets were also downloaded from the GEO database, and probes matching with multiple gene symbols were eliminated, while the mean values were calculated for gene symbols corresponding to multiple probes. The differentially expressed microRNAs (DEMis) and the differentially expressed mRNAs (DEMs) between HSCR and control samples in each dataset were identified by the Linear Models for Microarray Data (limma) package V.3.46.0^24^ with the cut-off criteria of |log2 fold change|>0.5 and p value of <0.05. The Venn diagram was used to obtain the common DEMs between the two mRNA microarray datasets.

### Hub DEMi identification

The miRNA similarity database (MISIM V.2.0, http://www.lirmed.com/misim/)^25^ was searched to recognize hub DEMis according to the MISIM V.2.0 Tutorial (http://www.lirmed.com/misim/Help).

### miRNA–target interaction investigation

The multiMiR package V.1.20.0^26^ was used to investigate the miRNA–target interactions. This package is a collection of miRNAs/targets from 14 external resources, including three validated miRNA–target databases (miRecords, miRTarBase, and TarBase) and eight predicted micRNA–target databases (DIANA-microT, ElMMo, MicroCosm, miRanda, miRDB, PicTar, PITA, and TargetScan), and so on, which can be used to retrieve all the validated and predicted target genes of a given miRNA, and all the validated and predicted miRNA–target interactions between a set of given miRNAs and mRNAs. Meanwhile, the top ten ranked miRNA–target couples were identified by Maximal Clique Centrality (MCC) algorithm via Cytoscape software V.3.8.2.

### Protein–protein interaction (PPI) network analysis

All the target genes of hub DEMis identified previously were uploaded to the STRING database V.11.5 (https://www.string-db.org/)^27^ to construct the PPI network. Confidence of >0.4 was set as the screening criteria. The PPI network was subsequently reconstructed and visualized by Cytoscape software V.3.8.2. The robust target genes were subsequently screened out using the cytoHubba plugin,^28^ which investigates the most important nodes in the PPI network with several topological analysis algorithms.

### Robust Rank Aggregation (RRA) analysis

To minimize the bias and inconsistencies, we integrated the top 20 ranked genes in the PPI network calculated by eight different topological analysis algorithms (MCC, MNC, EPC, EcCentricity, DMNC, Degree, Closeness, and BottleNeck method), and the RRA package V.1.1^29^ was adopted to identify the robust target genes. The score in the RRA analysis result indicated the ranking degree of each gene in the gene list, and the genes with a score of <0.05 were considered as the robust target genes.

### Functional and pathway enrichment analyses

Gene Ontology (GO) and Kyoto Encyclopedia of Genes and Genomes (KEGG) enrichment analyses were used to investigate the biological process, cellular component, molecular function, and involved pathways of selected genes, which were performed with the clusterProfiler R package V.4.6.0.^30^ The GO/KEGG terms with an adjusted p value of <0.05 were considered statistically significant and were visualized via the ggplot2 R package V.3.3.3.

### TF–miRNA–mRNA regulatory network analysis

The TF–miRNA regulations database (TransmiR V.2.0, http://www.targetscan.org/vert_72/)^31^ was searched to collect TFs of given miRNAs. Only the validated TF–miRNA interactions were included to construct the TF–miRNA–mRNA regulatory network, in which the key TRN regulon module was identified by the CytoHubba plugin.^28^ Moreover, the potential TF–miRNA interactions were further analyzed in the University of California Santa Cruz (UCSC) genome browser (https://genome.ucsc.edu/), and the TF–mRNA correlation in the colon was further analyzed in the Gene Regulatory Network Database (GRNdb, http://www.grndb.com/).^32^

### Diagnostic analysis of the key TRN regulons in HSCR

The ROC curve was obtained by GraphPad Prism software V.8.0.1 to assess the accuracy of each key TRN regulon as biomarkers in predicting HSCR. The machine learning based on the SVM method was used to establish an integrated diagnostic model followed by the ROC curve.

### Statistical analysis

Statistical analysis was performed by GraphPad Prism software V.8.0.1. Normally distributed data were presented as means±standard deviation (SD), and two-tailed Student’s t-test was applied to compare differences between groups. Statistical significance was set at a p value of <0.05.

## Results

### Microarray datasets and the workflow of this study

**Figure 1:**
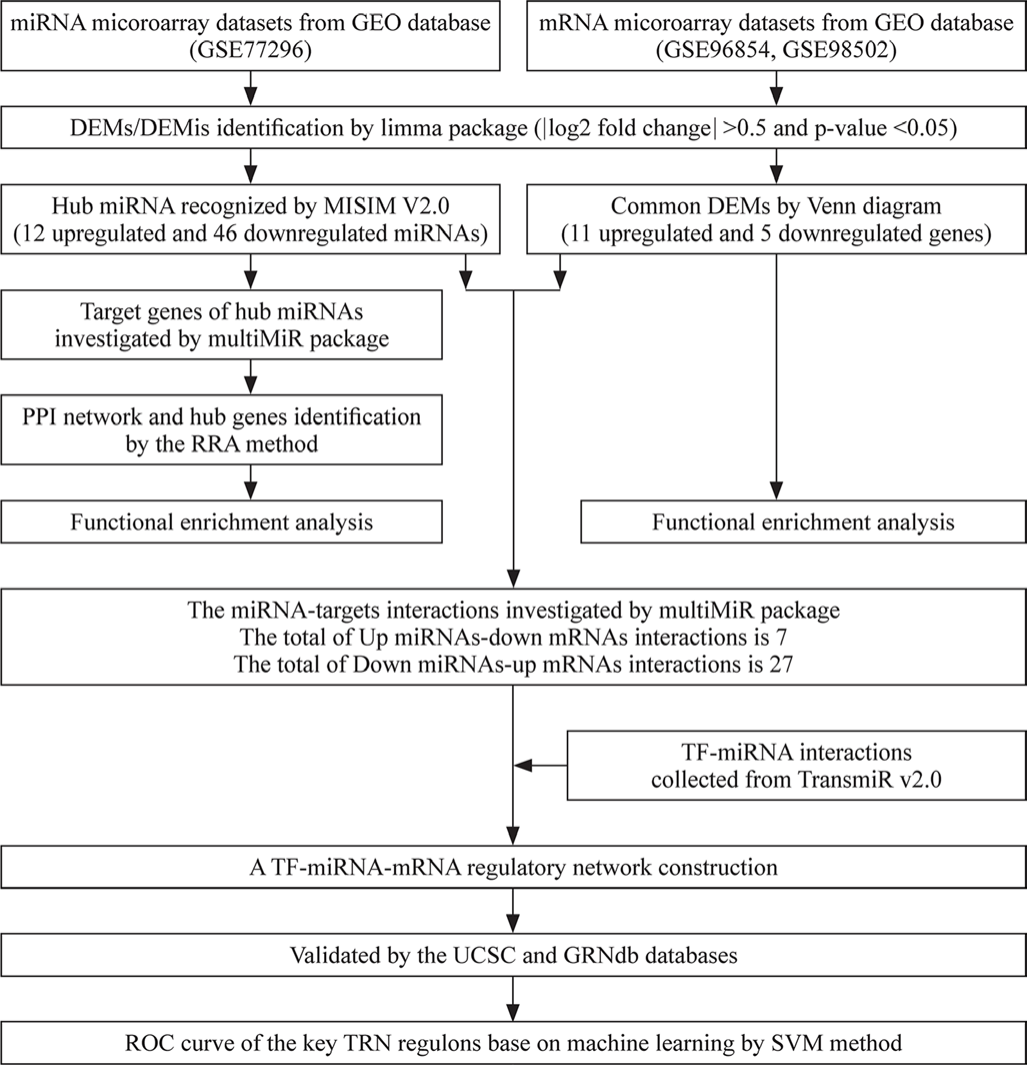
The whole study workflow. DEM, differentially expressed mRNA; DEMi, differentially expressed microRNA; GEO, Gene Expression Omnibus; GRNdb, Gene Regulatory Network Database; miRNA, microRNA; MISIM V.2.0, miRNA Similarity Database V.2.0; PPI, protein–protein interaction; ROC, receiver operating characteristic; RRA, Robust Rank Aggregation; SVM, support vector machine; TransmiR V.2.0, Transcription Factor Micro-RNA Regulations Database V.2.0; UCSC, University of California Santa Cruz; TRN, transcriptional regulatory network.

The microarray datasets derived from patients with HSCR were obtained from the GEO database. Only the databases with the normal subjects for control were included for further analysis, including two mRNA microarray datasets (GSE96854 and GSE98502) and one miRNA microarray dataset (GSE77296). The workflow of the study is shown in figure 1. Detailed information of the three datasets is shown in table 1.

### Identification of hub DEMis in HSCR

**Figure 2:**
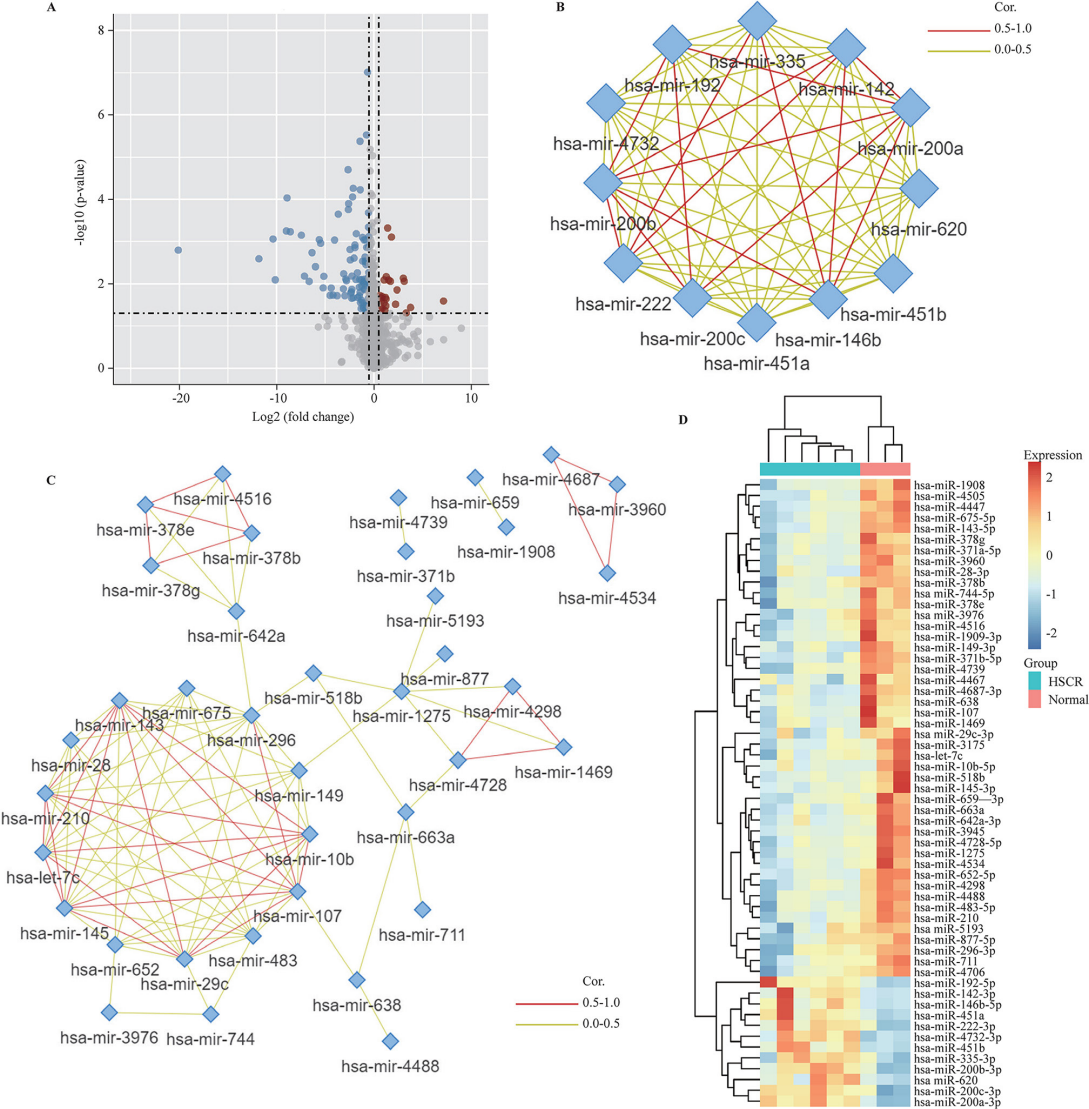
Identification of hub DEMis in HSCR. (A) Volcano plot of miRNA microarray dataset GSE77296. The 21 upregulated miRNAs are marked in red; the 83 downregulated miRNAs are marked in blue; and the gray dots represent miRNAs with no significant difference. Network of miRNAs interaction were searched in the MISIM V.2.0 to recognize upregulated (B) and downregulated (C) hub DEMis. (D) Heatmap diagram of the hub DEMis. DEMi, differentially expressed microRNA; HSCR, Hirschsprung disease; miRNA, microRNA; MISIM V.2.0, miRNA Similarity Database V.2.0.

The miRNA microarray dataset (GSE77296) was analyzed by the limma package to identify DEMis of the colon between patients with HSCR and healthy controls. When setting the cut-off criteria as follows: p value of <0.05 and |log2 fold change|>0.5, we obtained 104 DEMis (including 21 upregulated and 83 downregulated DEMis) (figure 2A). Then, we searched the miRNA similarity database (MISIM V.2.0, http://www.lirmed.com/misim/) to recognize hub DEMis, generating 12 upregulated (figure 2B) and 46 downregulated (figure 2C) miRNAs, all of which were illustrated as heatmap (figure 2D) and detailed in online supplemental table 1.

10.1136/wjps-2022-000547.supp1 Supplementary data

### Investigation and functional annotation of the genes targeted by hub DEMis

**Figure 3:**
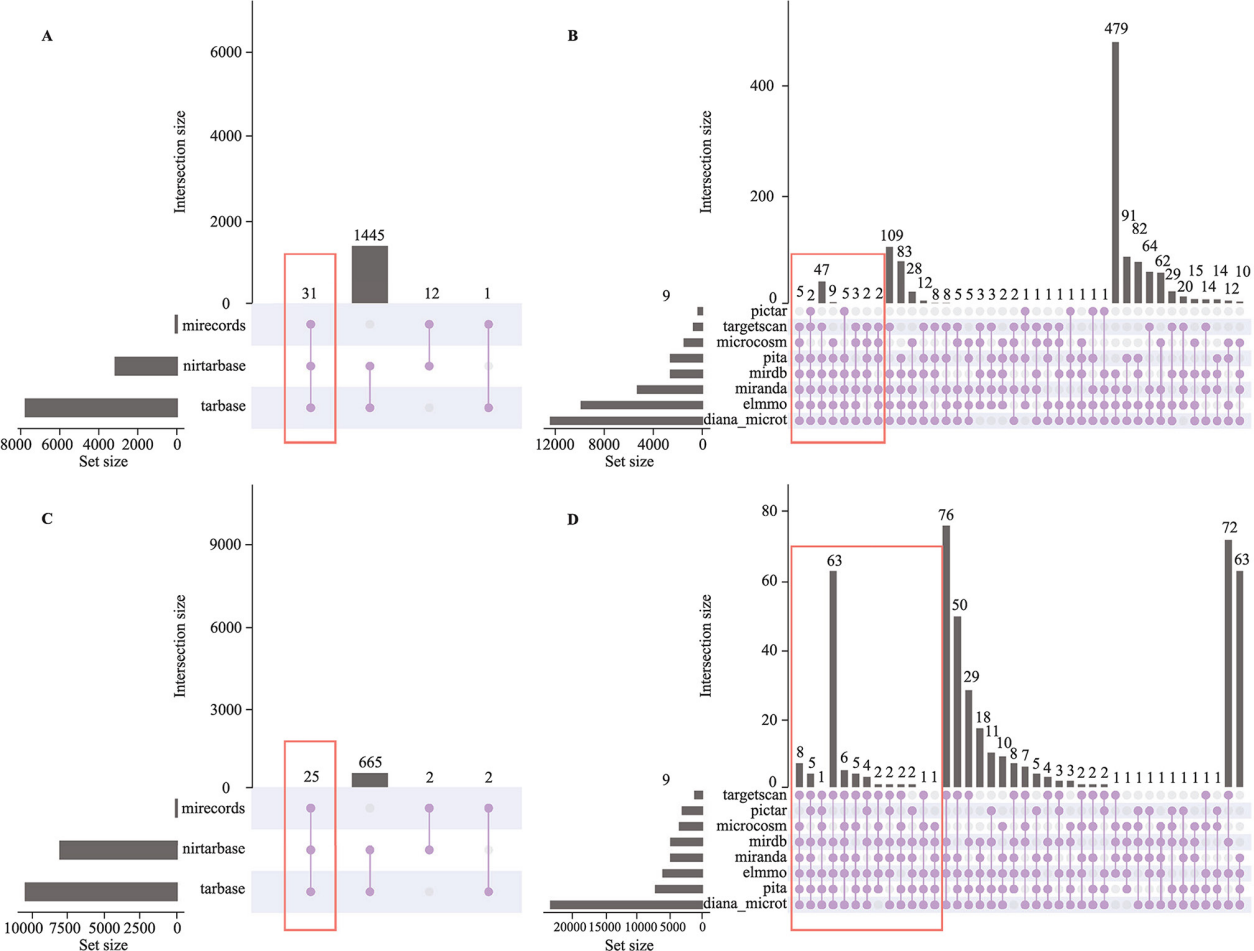
Investigation of the genes targeted by hub DEMis. The multiMiR package was used to investigate the miRNA–target interactions. Upset diagram of target genes of upregulated hub DEMis in validated (A) and predicted (B) miRNA–target databases. Upset diagram of target genes of downregulated hub DEMis in validated (C) and predicted (D) miRNA–target databases. The genes marked with red boxes were shared targets in three validated databases or at least six predicted databases. DEMi, differentially expressed microRNA; miRNA, microRNA.

The multiMiR package was used to investigate the genes targeted by hub DEMis. The target genes shared in three validated databases or at least six predicted databases were chosen in subsequent analysis, including 31 validated and 75 predicted target genes of upregulated miRNAs, while 25 validated and 102 predicted target genes of downregulated miRNAs (marked with a red box in figure 3).

**Figure 4:**
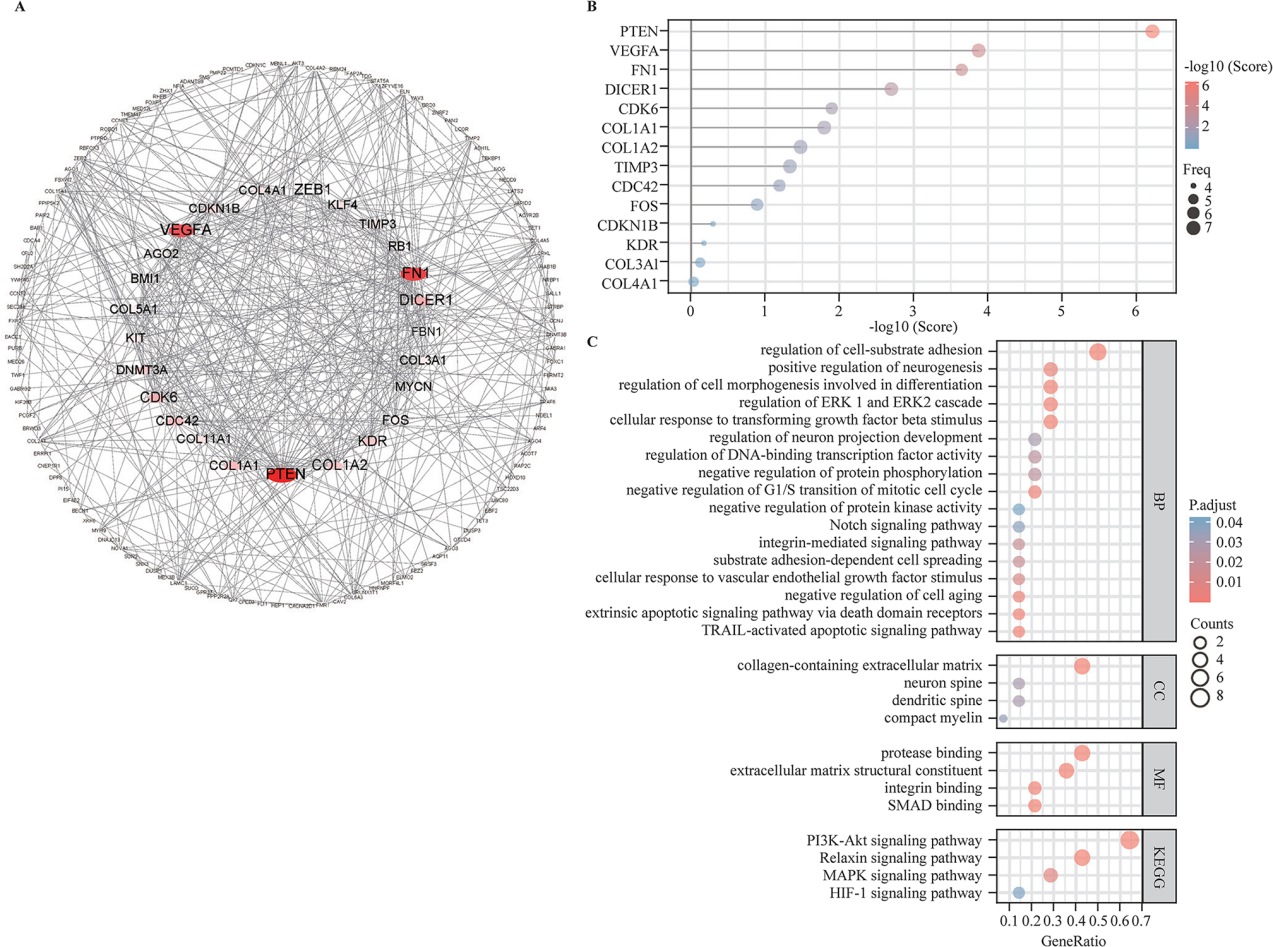
Functional annotation of the genes targeted by hub DEMis. (A) Whole PPI network with all target genes of hub DEMis; the bigger dots and deeper represent the higher degree. (B) The lollipop chart shows all robust target genes identified by the RRA method; the bigger dots represent the higher rank. (C) GO/KEGG functional enrichment analysis. BP, biological process; CC, cellular component; DEMi, differentially expressed microRNA; GO, Gene Ontology; KEGG, Kyoto Encyclopedia of Genes and Genomes; MF, molecular function; PPI, protein–protein interaction; RRA, Robust Rank Aggregation.

After removing duplicates, 197 target genes were uploaded to the STRING database (http://string.embl.de/) to perform PPI analysis. Then, to hide the disconnected nodes, the Cytoscape software was adopted to visualize the network (figure 4A). Robust target genes were subsequently screened out using the cytoHubba plugin, which investigates the most important nodes in the PPI network with several topological analysis algorithms. To improve the positive rate, the RRA method was used to integrate the top 20 ranked genes calculated by eight different topological analysis algorithms (MCC, MNC, EPC, EcCentricity, DMNC, Degree, Closeness, and BottleNeck), and a total of 14 genes were obtained accordingly (figure 4B). The upset diagram of the top 20 ranked genes from the eight algorithms is shown in online supplemental figure 1. Finally, GO/KEGG functional analysis was performed to explore the biological classifications of robust target genes in HSCR by the clusterProfiler package (figure 4C). GO enrichment analyses showed that the significantly enriched terms were related to the following: neurogenesis; cell cycle, apoptosis, differentiation, aging, and cell–substrate adhesion; protein phosphorylation; protein kinase activity; cellular response to transforming growth factor beta stimulus and vascular endothelial growth factor stimulus; DNA-binding TF activity, etc. In the KEGG pathway analysis, the significantly enriched terms were PI3K–Akt, mitogen-activated protein kinase (MAPK) (ERK1/2), notch, relaxin, and HIF-1 signaling pathway. RAS/MAPK and PI3K–Akt had been reported as the key signaling pathways in neurogenesis and neuroprotection^20 33–36^ and were related to RET and RET-regulating pathways in HSCR.^14^

Taken together, these data indicated that the hub DEMis and their target genes identified previously contributed to the pathogenesis of HSCR.

### Investigation and functional annotation of DEMs in HSCR

**Figure 5:**
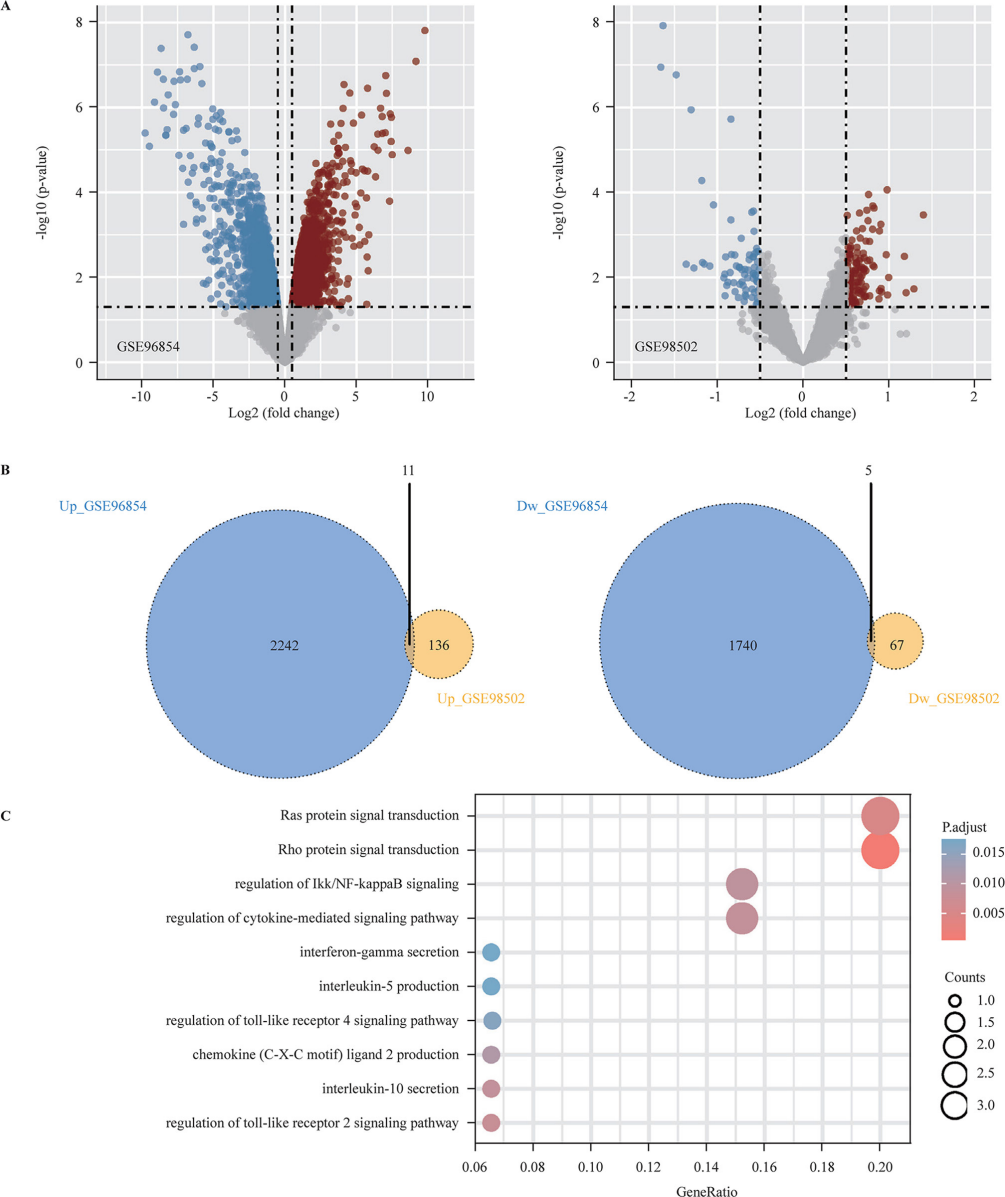
Investigation and functional annotation of the DEMs in HSCR. (A) Volcano plot of mRNA microarray datasets GSE96854 and GSE98502; the upregulated mRNAs are marked in red; the downregulated mRNAs are marked in blue; and the gray dots represent mRNAs with no significant difference. (B) Venn diagram demonstrates the common 11 upregulated and 5 downregulated DEMs. (C) GO/KEGG functional enrichment analysis. DEM, differentially expressed mRNA; GO, Gene Ontology; HSCR, Hirschsprung disease; KEGG, the Kyoto Encyclopedia of Genes and Genomes.

We further analyzed the other two mRNA datasets (GSE96854 and GSE98502) to identify the DEMs in the colon between patients with HSCR and healthy controls. When setting the cut-off criteria as follows: p value of <0.05 and |log2 fold change|>0.5, we obtained 3998 DEMs (including 2253 upregulated and 1745 downregulated DEMs) in GSE96854 and 219 DEMs (including 147 upregulated and 72 downregulated DEMs) in GSE98502 (figure 5A). The common DEMs in the two datasets (including 11 upregulated and 5 downregulated genes) (figure 5B) are detailed in table 2, which were significantly enriched in the GO/KEGG terms of Rho protein signal transduction, Ras protein signal transduction, IKappaB kinase (IKK)/nuclear factor kappa B (NF-κB), and cytokine-mediated signaling pathway (interferon-gamma, interleukin-5, interleukin-10, etc) (figure 5C). Various studies have shown that Rho/ROCK,^37–39^ RAS/MAPK,^20 33 40^ and IKK/NF-κB^20 41^ signaling played crucial roles in neurogenesis, which suggests the significant roles of the common DEMs in HSCR pathogenesis.

**Table 2:** Characteristics of the 16 common differentially expressed mRNAs

Symbol	Description	Ensembl	Regulation	Primary function
CA1	Carbonic anhydrase 1	ENSG00000133742	Up	Catalyzing the reversible hydration of carbon dioxide
ST3GAL4	ST3 beta-galactoside alpha-2,3-sialyltransferase 4	ENSG00000110080	Up	Participating in protein glycosylation
PAQR5	Progestin and adipoQ receptor family member 5	ENSG00000137819	Up	Plasma membrane progesterone (P4) receptor coupled to G proteins
IL1RL1	Interleukin 1 receptor like 1	ENSG00000115602	Up	The interleukin 1 receptor family involved in the function of helper T cells
F2RL1	F2R like trypsin receptor 1	ENSG00000164251	Up	The G-protein coupled receptor 1 family followed by PLC, MAPK, IKK/NF-κB, and Rho signaling
KCNN2	Potassium calcium-activated channel subfamily N member 2	ENSG00000080709	Up	Regulating neuronal excitability by contributing to the slow component of synaptic AHP
SLC36A4	Solute carrier family 36 member 4	ENSG00000180773	Up	A sodium-independent electroneutral transporter for amino acids
PIM3	Pim-3 proto-oncogene, serine/threonine kinasep	ENSG00000198335	Up	A proto-oncogene with serine/threonine kinase activity, regulating cell apoptosis
SORBS2	Sorbin and SH3 domain containing 2	ENSG00000154556	Up	The member of the Abelson family of non-receptor protein–tyrosine kinases
CRB1	Crumbs cell polarity complex component 1	ENSG00000134376	Up	Participating in photoreceptor morphogenesis in the retina
CHUK	Component of inhibitor of nuclear factor kappa B kinase complex	ENSG00000213341	Up	A component of a cytokine-activated protein complex as an inhibitor of NF-κB
ABCG5	ATP-binding cassette subfamily G member 5	ENSG00000138075	Down	Mediating Mg (2+)-dependent and ATP-dependent sterol transport across the cell membrane
C1orf115	Chromosome one open reading frame 115	ENSG00000162817	Down	Being associated with spastic paraplegia and autosomal recessive
EGFL6	EGF like domain multiple 6	ENSG00000198759	Down	A member of EGF repeat superfamily involved in the cell cycle, proliferation, and developmental processes
RND2	Rho family GTPase 2	ENSG00000108830	Down	A member of the Rho GTPase family, regulating neuronal morphology and endosomal trafficking
PGPEP1	Pyroglutamyl-peptidase I	ENSG00000130517	Down	A member of the peptidase C15 family

AHP, afterhyperpolarization; CHUK, conserved helix–loop–helix ubiquitous kinase; EGF, epidermal growth factor; IKK, IκB kinase; MAPK, mitogen-activated protein kinase; NF-κB, nuclear factor kappa B; PLC, phospholipase C; SH3, src homology.

### Analysis of TF–miRNA–mRNA regulatory network

**Figure 6:**
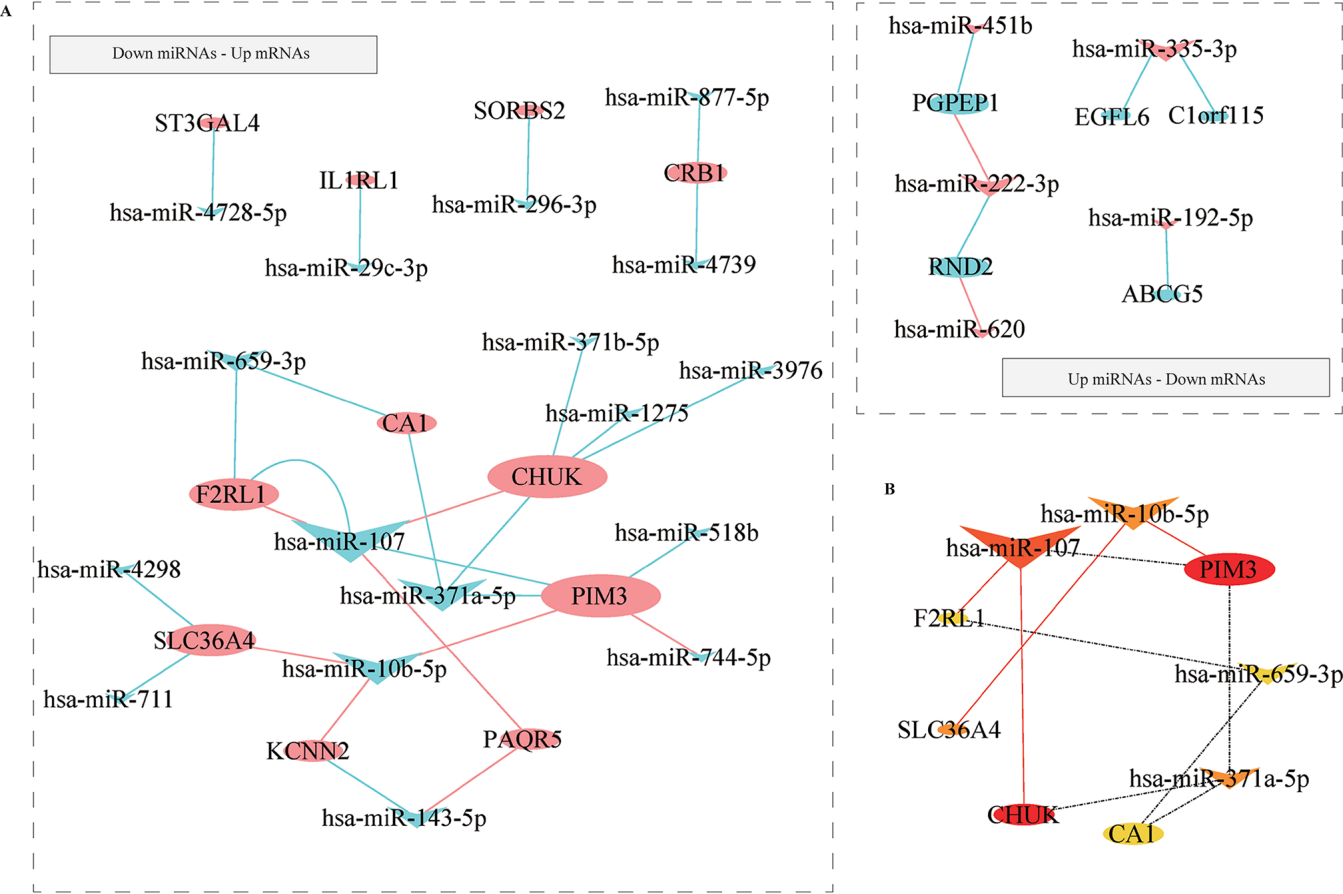
Investigation of miRNA–target interactions. (A) miRNA–target interactions. The miRNAs are marked as diamonds, and mRNAs are marked as ellipses; upregulated genes are marked in red, while the downregulated genes are marked in green; bigger nodes indicate the higher degree; red and green lines represent the validated and predicted miRNA–target couples, respectively. (B) Top 10 ranked miRNA–target couples identified by MCC algorithm. The miRNAs and mRNAs are marked as diamonds and ellipses, respectively; sizes and colors of nodes represent the degree in the network. miRNA, microRNA; MCC, Maximal Clique Centrality.

For a robust miRNA–target interaction, we investigated the hub DEMis and the DEMs shared in two databases by the multiMiR package. A total of 34 miRNA–target couples were identified, including 7 upregulated miRNA-downregulated mRNAs (2 validated and 5 predicted miRNA–target couples) and 27 downregulated miRNA-upregulated mRNA interactions (8 validated and 19 predicted miRNA–target couples) (figure 6A), all of which are detailed in online supplemental table 2. The top 10 ranked miRNA–target couples were identified by the MCC algorithm (figure 6B).

**Figure 7:**
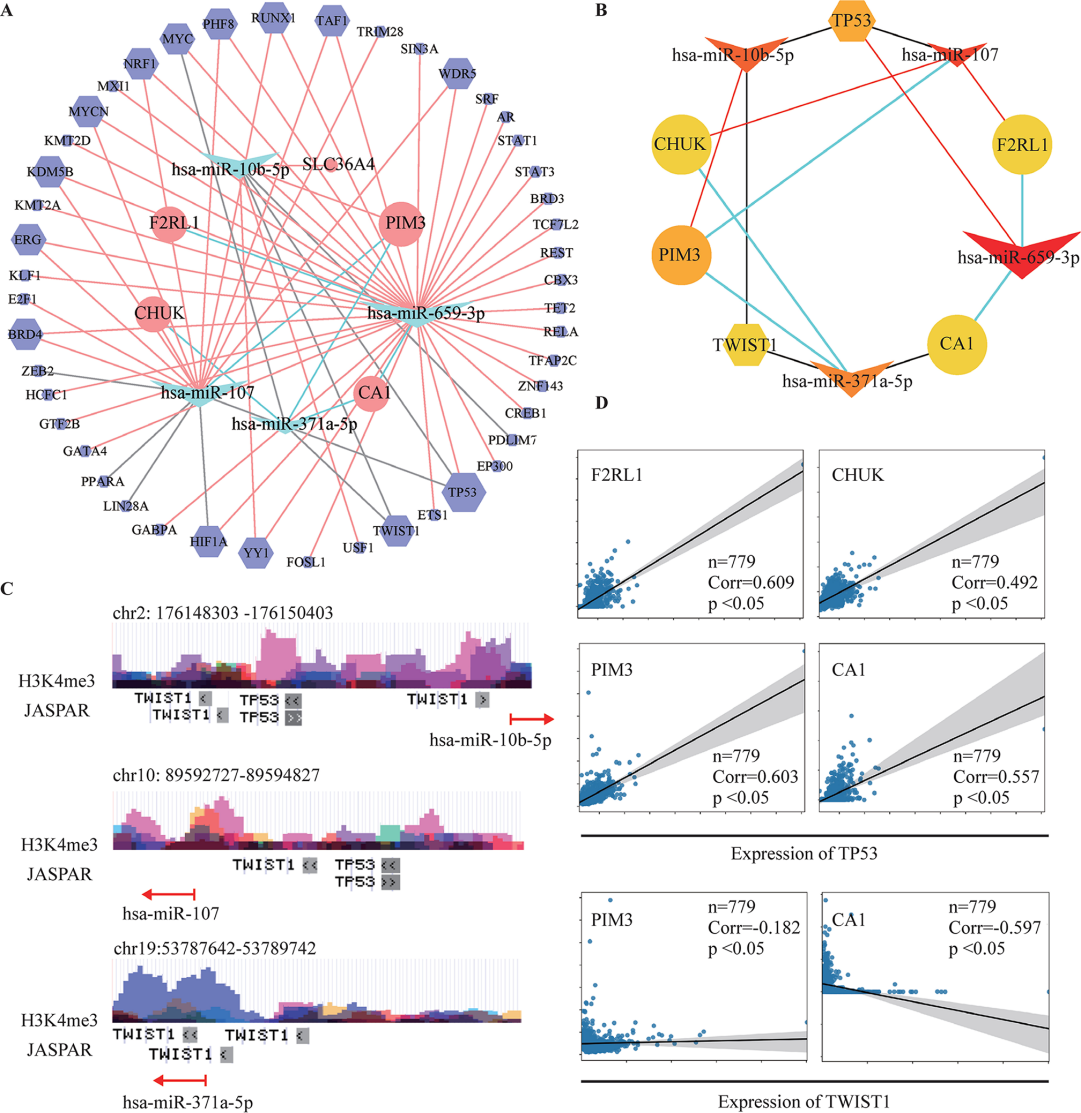
Analysis of TF–miRNA–mRNA network. (A) The TF–miRNA–mRNA network. The miRNAs, mRNAs, and TFs are marked as diamonds, ellipses, and octagons, respectively; upregulated genes are marked in red, while the downregulated genes are marked in green; bigger nodes indicate the higher degree; red, gray, and green lines represent the validated, reported, and predicted connections, respectively. (B) Key modules of TF–miRNA–mRNA network identified by cytoHubba. The miRNAs, mRNAs, and TFs are marked as diamonds, ellipses, and octagons, respectively; sizes and colors of nodes represent the degree in the network. (C) Promoter region analysis of miRNA genes in UCSC genome browser (https://genome.ucsc.edu/). Higher level of H3K4me3 methylated modification represents the more reliable TF–target relationship. (D) Correlation analysis of TFs and miRNA-targeted mRNAs in the GRNdb (http://www.grndb.com/). GRNdb, Gene Regulatory Network Database; miRNA, microRNA; TF, transcription factor; UCSC, University of California Santa Cruz.

Then, we searched the TF–miRNA regulations database (TransmiR V.2.0, http://www.cuilab.cn/transmir) for the TFs that target the miRNAs in figure 6B. Only the validated TF–miRNA interactions were included to construct the TF–miRNA–mRNA regulatory network (figure 7A). The cytoHubba was used to identify the key modules (figure 7B), which included 2 TFs (*TP53* and *TWIST1*), 4 miRNAs (*has-miR-107*, *has-miR-10b-5p*, *has-miR-659-3p*, and *has-miR-371a-5p*), and 4 mRNAs (*PIM3*, conserved helix–loop–helix ubiquitous kinase (*CHUK*), *F2RL1*, and *CA1*). Finally, the potential TF–miRNA interactions were further analyzed in the UCSC genome browser (https://genome.ucsc.edu/) (figure 7C), that is, the promoter region analysis of miRNA genes, showing that a higher level of H3K4me3 methylated modification represents the more reliable TF–target relationship. The correlation analysis of TFs and miRNA-targeted mRNAs in the colon was further analyzed in the GRNdb (http://www.grndb.com/) (figure 7D).

### Diagnostic value of the key TRN regulons as biomarkers in HSCR

**Figure 8:**
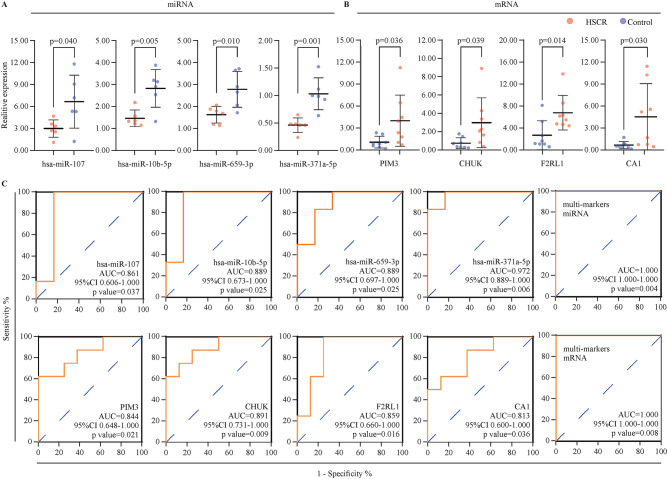
Diagnostic value of the key TRN regulons as biomarkers in HSCR (A, B) Relative expression of the eight key TRN regulons (four miRNAs and four mRNAs) in HSCR. (C) ROC curve of the eight key TRN regulons and the integrated diagnosis model based on machine learning by SVM method. AUC, area under the curve; HSCR, Hirschsprung disease; miRNA, microRNA; ROC, receiver operating characteristic; SVM, support vector machine; TRN, transcriptional regulatory network; 95% CI, 95% confidence interval.

The gold standard for the diagnosis of HSCR is rectal mucosal aspiration biopsy and pathological diagnosis, which are commonly invasive and difficult to perform.^1 2^ The relative expression of the key TRN regulons (*has-miR-107*, *has-miR-10b-5p*, *has-miR-659-3p*, *has-miR-371a-5p*, *PIM3*, *CHUK*, *F2RL1*, and *CA1*) were visualized as boxplots (figure 8A, B). To investigate the diagnostic value of these regulons in HSCR, the ROC curve was used, which showed that all eight regulons had area under the curve (AUC) values more than 0.8, indicating a strong diagnostic value (figure 8C). For better diagnosis prediction, these eight regulons were integrated to establish a multimarker diagnosis model based on machine learning by the SVM method. The ROC curve showed that the multimarker models could effectively predict HSCR (AUC=1.00) (figure 8C).

## Discussion

The transplantation of ENCCs to induce enteric neurogenesis is a potential radical strategy for HSCR while generating insufficient efficacy. It may due to the complex genes regulatory to ENCCs in children with HSCR.^1 5 9^ Although many genes have been identified to be associated with HSCR,^2 11^ such as *RET*, *EDNRB*, *RARB*, *GATA2*, and *SOX10*, which commonly regulate ENCCs during the development of ENS, how the TRN contributes to HSCR pathogenesis remains to be investigated. This study identified a potential TF–miRNA–mRNA network, including the key regulons of two TFs (*TP53* and *TWIST1*), four miRNAs (*has-miR-107*, *has-miR-10b-5p*, *has-miR-659-3p*, and *has-miR-371a-5p*), and four mRNAs (*PIM3*, *CHUK*, *F2RL1*, and *CA1*), that can help enrich the connotation of HSCR pathogenesis and diagnosis and provide new horizons for treatment.

Many miRNAs have been reported to be related to HSCR,^13–15^ including *miRNA-206/SDPR*,^16 42^ *miR-146b-5p/RET*,^17^ and *miR-181a/RAP1B*.^18^ In this study, we found that *has-miR-107*, *has-miR-10b-5p*, *has-miR-659-3-p*, and *has-miR-371a-5p* were related to HSCR and exerted good diagnostic value. As reported, *has-miR-107* regulated Wnt/β-catenin signaling^43^ and attenuated neurotoxicity induced by 6-hydroxydopamine.^44^ *MiR-10b-5p* contributed to neurodegenerative disease, diabetes with dysfunction of interstitial Cajal cells, and neuroprotection for hippocampal neuronal cells.^45–48^ In cancer diseases, *miR-659–3p* and *miR-371a-5p* could regulate tumor progression and were associated with chemotherapy resistance.^49–53^ Novel research has shown that specific miRNAs in serum or plasma exosomal were identified to have good diagnostic value in HSCR.^54 55^ As mentioned previously, the miRNAs identified in this study had AUC values of more than 0.8 and remained unclear so far in HSCR, which provided new cues for future biomarker study of HSCR treatment and diagnosis.

As reported, approximately 50% of familial and 20% of patients with sporadic HSCR had *RET* expression abnormalities; 5% of patients had *EDNRB* variations, while 4% of patients had *SOX10* variations. It seems to be difficult to diagnose HSCR by any one of the known pathogenic genes due to the complex non-Mendelian inheritance. In this study, we constructed a potential TF–miRNA–mRNA network, of which a key module with four functional genes (*PIM3*, *CHUK*, *F2RL1*, and *CA1*) was identified. Based on the key regulons, we constructed a multimarker model by the SVM method, which had an AUC equal to 1 to effectively predict HSCR. It has been reported that *PIM3*, a proto-oncogene with serine/threonine kinase activity, could regulate cell migration and apoptosis via PI3K–AKT, p38, or Rho GTPase signaling,^56–58^ and was related to demyelinating disease.^59^ Inhibitor-κB kinase α, which is encoded by the *CHUK* gene, was recognized to regulate NF-κB activity^60 61^ and involved the differentiation of mouse embryonic neuroectoderm. *F2RL1* was reported as the key protease-activated receptor to stimulate neuronal repair after ischemic injury.^62 63^ The GO/KEGG annotations of carbonic anhydrase 1 (*CA1*) were carbonate dehydratase activity, hydrolyase activity and interleukin-12 family signaling. At present, all the aforementioned genes were still unclear but relevant to neuropathies, especially HSCR.

As reported, the development and functional maturity of ENS is regulated by complex mechanisms, which largely depend on the potential of ‘seed’ ENCCs and their compatibility with the intestinal microenvironment ‘niche’.^64 65^ The genetic factors, such as gene mutations (including *RET*, *EDNRB*, *RARB*, *GATA2*, *SOX10*, *PHOX2B*, etc)^2 65^ and signaling pathway disorders (including PI3K–Akt, MAPK, IKK/NF-κB, Rho/ROCK, etc), determine the inborn developmental potential of ENCCs. Meanwhile, the critical role of intestinal microenvironment, such as glial cell line-derived neurotrophic factor, 5-hydroxytryptamine, semaphorins, neuregulin 1, the extracellular matrix molecules (collagen, laminin, proteoglycans, etc),^65^ postnatal intestinal flora colonization, and their metabolites,^66^ has been gradually recognized. Although the functional annotation of the TRN regulons mentioned previously appeared to be associated with the signaling pathways in neurogenesis and neuroprotection, which suggests the significant roles in HSCR pathogenesis, how the TRN regulons regulate the ENCCs and interact with these intestinal microenvironment niche remain to be further investigated.

In conclusion, this study provided a potential TF–miRNA–mRNA network based on integrated analysis of three microarray datasets. ROC analysis based on the SVM method revealed a strong diagnostic value of the key TRN regulons, which can help enrich the connotation of HSCR pathogenesis and diagnosis and provide new horizons for further study. However, due to the limited datasets of HSCR, an integrated model containing miRNAs and mRNA to predict HSCR was unavailable. Moreover, further validated experiments with cells and animals were extensible.

## Data Availability

Data are available in a public, open access repository. Publicly available datasets (GSE96854, GSE98502, and GSE77296) were analyzed in this study. All the datasets can be found in the Gene Expression Omnibus database (https://www.ncbi.nlm.nih.gov/geo/).
