# Supplementary material for: Identifying the potential transcriptional regulatory network in Hirschsprung disease by integrated analysis of microarray datasets
Source: World J Pediatr Surg. 2023 Apr 17;6(2):e000547. doi: 10.1136/wjps-2022-000547 (PMC10111925; doi:10.1136/wjps-2022-000547)
Supplement: Supplementary data [file wjps-2022-000547supp001.pdf]

Supplement Figure 1

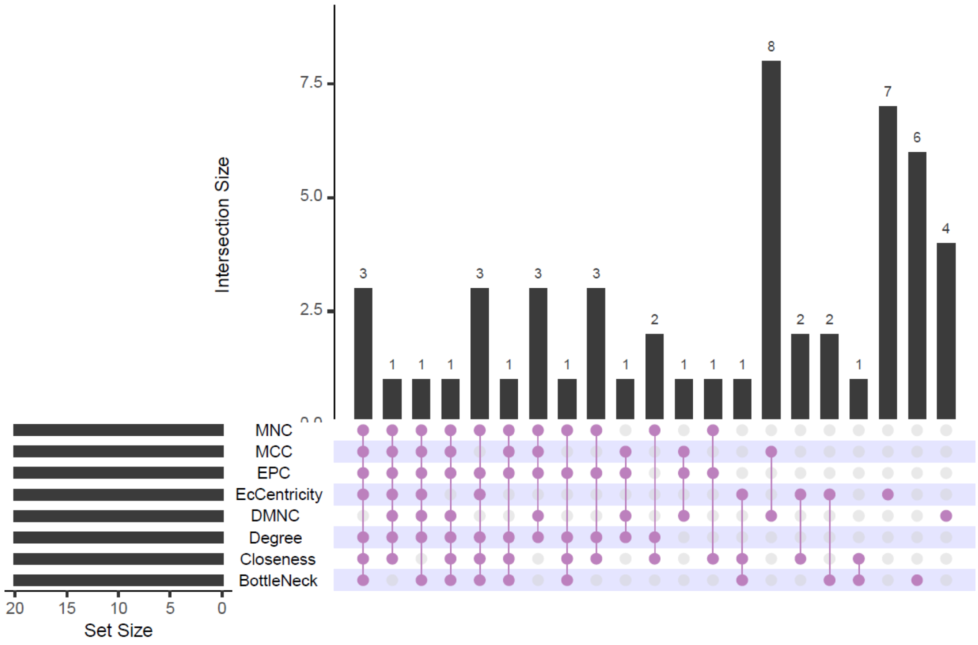

Upset diagram of the top 20 ranked target genes calculated by 8 different topological analysis algorithms (MCC, MNC, EPC, EcCentricity, DMNC, Degree, Closeness and BottleNeck).

Supplement Table 1. The hub differentially expressed miRNAs

	miRNAs	logFC	p.Value
**up regulated**	hsa-miR-142-3p	21.06360449	0.045485
	hsa-miR-451a	7.169953881	0.025696
	hsa-miR-335-3p	3.332957948	0.049159
	hsa-miR-200b-3p	3.135328816	0.008717
	hsa-miR-200c-3p	3.047008694	0.007391
	hsa-miR-222-3p	2.231782958	0.030586
	hsa-miR-200a-3p	1.812422389	0.000788
	hsa-miR-4732-3p	1.702820187	0.008739
	hsa-miR-192-5p	1.178932998	0.020877
	hsa-miR-620	1.124317865	0.042277
	hsa-miR-146b-5p	0.860147065	0.038711
	hsa-miR-451b	0.764642318	0.021567
**down regulated**	hsa-miR-744-5p	-11.81585744	0.002565
	hsa-miR-1908	-9.000858408	0.000565
	hsa-miR-371b-5p	-8.923302546	0.000093
	hsa-miR-3960	-7.443943512	0.000709
	hsa-miR-4516	-7.126796129	0.006600
	hsa-miR-483-5p	-5.995398466	0.003946
	hsa-miR-149-3p	-5.462608298	0.001102
	hsa-miR-4467	-5.202627466	0.012538
	hsa-miR-1275	-5.137464615	0.006675
	hsa-miR-107	-4.494242201	0.018847
	hsa-miR-4687-3p	-4.03278594	0.000927
	hsa-miR-3175	-3.771208079	0.019254
	hsa-miR-4447	-3.651482831	0.000226
	hsa-miR-3976	-3.126512761	0.013214
	hsa-miR-1469	-2.976793679	0.019220
	hsa-miR-675-5p	-2.617105087	0.000175
	hsa-miR-4739	-2.418738215	0.002744
	hsa-miR-711	-2.260366563	0.013551
	hsa-miR-663a	-2.248958453	0.006601
	hsa-miR-4505	-2.183331826	0.000088
	hsa-miR-143-5p	-2.119258492	0.000055
	hsa-miR-638	-2.033734606	0.002483
	hsa-miR-5193	-1.651110747	0.021133
	hsa-miR-642a-3p	-1.499262182	0.012040
	hsa-miR-29c-3p	-1.412196901	0.016067
	hsa-miR-652-5p	-1.395518706	0.000060
	hsa-miR-10b-5p	-1.30702971	0.038645
	hsa-miR-4534	-1.255197089	0.008018
	hsa-miR-518b	-1.240372755	0.011314
	hsa-miR-4488	-1.158185029	0.000800
	hsa-miR-659-3p	-1.144956015	0.027727
	hsa-miR-145-3p	-1.09061667	0.009047
	hsa-miR-877-5p	-1.057007492	0.036342
	hsa-miR-378e	-1.028578655	0.008900

	hsa-miR-210	-1.012818082	0.000916
	hsa-miR-378b	-0.987183738	0.007846
	hsa-miR-4728-5p	-0.9865354	0.013719
	hsa-miR-3945	-0.966438711	0.010018
	hsa-miR-378g	-0.85364609	0.003056
	hsa-let-7c	-0.773019629	0.041752
	hsa-miR-28-3p	-0.734677439	0.001378
	hsa-miR-296-3p	-0.730205476	0.032571
	hsa-miR-1909-3p	-0.570267623	0.007085
	hsa-miR-4706	-0.559209286	0.009662
	hsa-miR-371a-5p	-0.552838476	0.000209
	hsa-miR-4298	-0.51530379	0.000770

Supplement Table 2. The miRNA-mRNA interactions identified by *multiMiR* package

miRNA	regulation	confidence	target mRNAs
hsa-miR-222-3p	up	validated	KIT / CDKN1B / CDKN1C / FOS / PPP2R2A / STAT5A / TIMP3
		predicted	CDKN1B / GABRA1 / HECTD2 / PCMTD1 / DMRT3 / TSC22D3 / KIT / ZFYVE16 / MIA3 / KDR / ADAM22 / MYLIP / ARF4 / FMR1 / VAPB / KIF16B / MIER3 / DPP8 / FAM214A / SYT10
hsa-miR-200a-3p	up	validated	ZEB2 / ZEB1 / BAP1 / KLHL20 / PTPRD / ELMO2 / WDR37
		predicted	DUSP3 / ANP32E / DNAJC13 / BRD3 / MBNL1 / RBM24 / TADA1 / FOXC1 / ACOT7 / RHEB / HNRNPF
hsa-miR-200b-3p	up	validated	ZEB1 / BAP1 / KLHL20 / PTPRD / ELMO2 / WDR37 / ERRFI1 / BMI1
		predicted	CFL2 / DUSP1 / NOG / SEC23A / RAP2C / NRBP1 / CNEP1R1 / YWHAG / HS3ST1 / NOVA1 / TFAP2A / FEZ2 / FN1 / RAB21
hsa-miR-200c-3p	up	validated	ZEB1 / BAP1 / KLHL20 / PTPRD / ELMO2 / WDR37 / ERRFI1
		predicted	MARCKS / ZEB2 / MMD / DUSP1 / CFL2 / CCNJ / SEC23A / RAP2C / FOXF1 / PDIK1L / TIMP2 / RBFOX3 / NOG / HS3ST1 / YWHAG / CNEP1R1 / NRBP1 / FLI1 / ZCCHC24 / FEZ2 / CHMP5 / TFAP2A / FN1 / RAB21 / QKI
hsa-miR-192-5p	up	validated	WNK1 / RB1
hsa-miR-142-3p	up	predicted	SUCO / ASH1L / LCOR
hsa-miR-146b-5p	up	predicted	TRAF6 / STRBP
hsa-miR-107	down	validated	BACE1 / AGO1 / AGO2 / AGO3 / CCNE1 / CDK6 / CDCA4 / RAB1B / CRKL / PLAG1 / DICER1
		predicted	ANO3 / MED26 / CACNA2D1 / ZHX1 / RBM24 / DICER1 / ZFPM2 / PCGF2 / ZNRF2 / AQP11 / GABRG2 / UNC80 / SALL1 / FERMT2 / JAKMIP2 / RNF38 / AGO4 / CAB39 / FBXW7 / SUN2 / NDEL1 / NFIA / TBKBP1 / CEP85L / TMEM47 / MYH9 / CPEB3 / PURB / VAV3 / ZBTB10 / FAM81A / RUNX1T1 / NEDD9 / PPIP5K2 / LATS2 / SH2D2A / GPCPD1 / TSPAN5 / TWF1 / DYRK2 / SNX3 / ACVR2B
hsa-miR-29c-3p	down	validated	DNMT3A / DNMT3B / COL3A1 / COL4A1 / COL15A1 / LAMC1 / TDG / COL1A1 / COL1A2 / COL4A2 / FBN1 / CDC42
		predicted	SESTD1 / TMEM183A / IFI30 / ELN / PAN2 / COL2A1 / COL6A3 / KIF26B / FOXJ2 / COL3A1 / COL4A1 / ROBO1 / COL4A5 / IREB2 / HBP1 / MEX3B / PMP22 / GPATCH2 / ADAMTS9 / LYSMD1 / BACH2 / ANKRD13B / ISG20L2 / GPR37 / BRWD3 / PDIK1L / COL1A2 / COL11A1 / TET1 / PI15 / CRISPLD1 / PTEN / CAV2 / COL5A1 / DNMT3A / OTUD4 / MYCN / MORF4L1 / REV3L / XKR6 / GPX7 / TIMM8B / CCNT2 / FRAT2 / MED12L / SMS / AKT3 / CCNYL1 / VEGFA / TET3 / JARID2 / PAIP2 / EIF4E2
hsa-miR-10b-5p	down	validated	HOXD10 / KLF4
		predicted	EBF2
hsa-miR-371a-5p	down	predicted	BTG3 / SRSF3 / SLC25A33 / BECN1
hsa-miR-877-5p	down	predicted	FXR2
hsa-miR-28-3p	down	predicted	SLC26A3
